# Vapor-Phase Dicarboxylic Acids and Anhydrides Drive Depolymerization of Polyurethanes

**DOI:** 10.1021/acsmacrolett.4c00008

**Published:** 2024-03-28

**Authors:** Baoyuan Liu, Zach Westman, Kelsey Richardson, Dingyuan Lim, Alan L. Stottlemyer, Paul Gillis, Christopher S. Letko, Nasim Hooshyar, Vojtech Vlcek, Phillip Christopher, Mahdi M. Abu-Omar

**Affiliations:** † Department of Chemistry and Biochemistry, University of California, Santa Barbara, California 93117, United States; ‡ Department of Chemical Engineering, University of California, Santa Barbara, California 93117, United States; § The Dow Chemical Company, Midland, Michigan 48642, United States; ∥ The Dow Chemical Company, Herbert H Dowweg 5, Hoek 4542 NH, The Netherlands

## Abstract

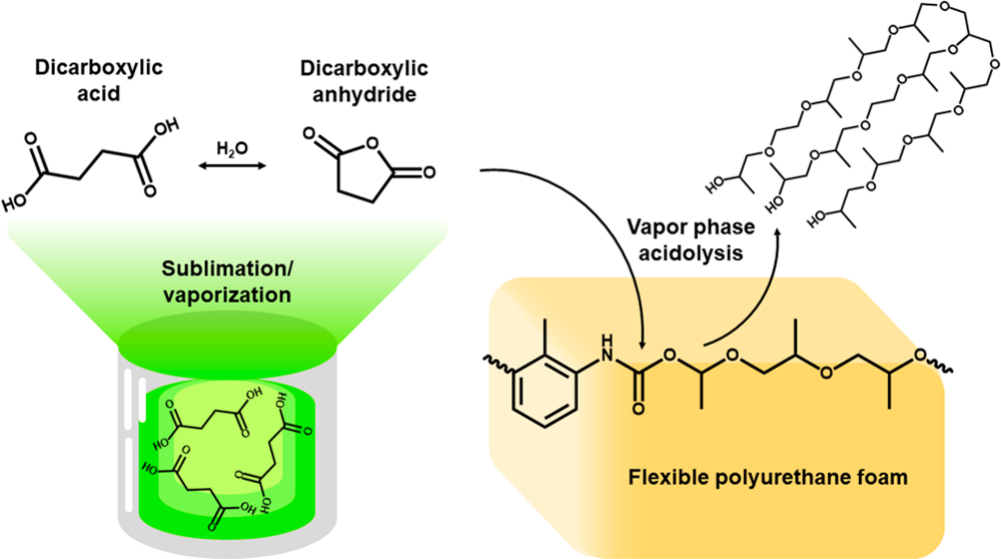

Polyurethane (PU) is the sixth most used plastic in the world. Because many PU derived materials are thermosets and the monomers are valuable, chemical recycling to recover the polyol component is the most viable pathway to utilizing postconsumer PU waste in a closed-loop fashion. Acidolysis is an effective method to recover polyol from PU waste. Previous studies of PU acidolysis rely on the use of dicarboxylic acid (DCA) in high temperature reactions (>200 °C) in the liquid phase and result in unwanted byproducts, high energy consumption, complex separations of excess organic acid, and an overall process that is difficult to scale up. In this work, we demonstrate selective PU acidolysis with DCA vapor to release polyol at temperatures below the melting points of the DCAs (<150 °C). Notably, acidolysis with DCA vapor adheres to the principles of green chemistry and prevents in part esterification of the polyol product, eliminating the need for additional hydrolysis/processing to obtain the desired product. The methodology was successfully applied to a commercial PU foam (PUF) postconsumer waste.

PU is the primary component in many consumer products, including mattresses and foams, and is generally produced through the reaction of relatively large (molecular weight >1000 amu) polyols and isocyanates. Acidolysis is effective for decomposing PU back to the starting polyol via reaction between the acid and urethane/urea linkages in PU, effectively enabling closed-loop molecular recycling of PU as polyols are ∼60–70% of the PU by mass.^1^ In addition to polyol, acidolysis products include amides, amine salts, carbon dioxide (CO_2_), water (H_2_O), and oligoureas.^2^ PU acidolysis with DCAs (e.g., adipic acid (AA), succinic acid (SA), or phthalic acid (PA)) is typically carried out at elevated temperatures, between 190–230 °C, and on time scales of several hours.^3−6^ The high temperatures are used because: (1) it has been assumed that liquid DCAs are required to react with solid PU and their melting points (*T*_m_) are AA *T*_m_ = 152 °C, SA *T*_m_ = 186 °C, and PA *T*_m_ = 207 °C, and (2) PU polymers themselves are thermally stable up to ≥200 °C. Disadvantages of elevated-temperature PU acidolysis include: (1) formation of undesired byproducts, (2) side reactions between the polyol product and DCA to produce esters that have to be subsequently hydrolyzed, and (3) large energy requirements. In this work, we report previously unrealized acidolysis driven by sublimation of DCAs and/or vaporization of their corresponding anhydrides, enabling PU acidolysis to polyols at moderate temperatures below the *T*_m_ of DCAs and as low as 140 °C. The new methodology avoids the formation of undesirable byproducts and eliminates the need for excess DCA or solvent. Investigations of the corresponding anhydrides as reactants and the effect of water provided insight into the transport mechanism that makes PU acidolysis feasible at temperatures below the *T*_m_ of the organic acid.

Both model and end-of-life (EOL) PU foam (PUF) were used as representative materials to study PU acidolysis with maleic acid (MA), SA, PA, and AA. The model PUF (provided by The Dow Chemical Company) was synthesized by reacting toluene diisocyanate (TDI) with VORANOL 8316 poly(ether polyol) (virgin polyol, Supporting Information (SI), Scheme S-1) and is an open cell, flexible foam. The corresponding acid anhydrides were also investigated, as they can be produced from DCAs at elevated temperatures, often have higher vapor pressures than the parent DCAs, and can readily convert back to acids in the presence of water. Scheme 1 describes the acidolysis reaction between DCAs and PUF to produce recycled polyol (repolyol), amide, CO_2_, and H_2_O.

**Scheme 1:**
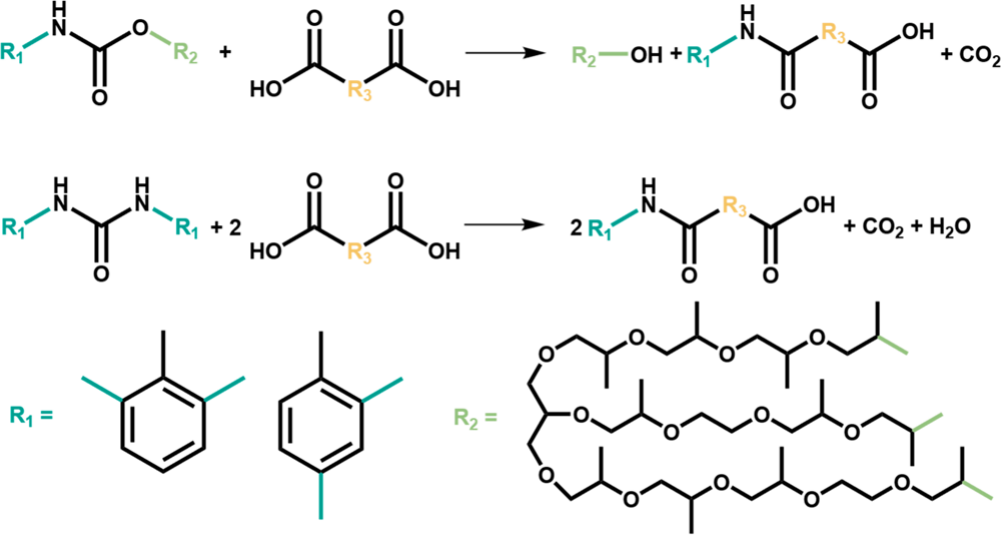
PU Acidolysis of Urethane (Top) and Urea (Bottom) with DCA to Product Amides, Repolyol, CO_2_, and H_2_O as Products at Elevated Temperature The model foam studied herein was composed of toluene diisocyanate (TDI, R_1_) and a polyether polyol (R_2_).

We previously demonstrated facile liquid-phase PU acidolysis with excess MA in the melt phase at 175 °C (Figure S-1).^7^ The fast rate of PU decomposition at 175 °C (<10 min for quantitative polyol ester formation) suggested that low temperature (<150 °C) acidolysis might be feasible. Low-temperature acidolysis reactions with PA and SA were carried out by physically mixing DCA solids with preground model PUF solid (in 3:1 DCA:PUF mass ratios) in a round-bottom flask and heating the flask to 140 °C in an oil bath. The progression of the acidolysis reaction was monitored via measurement of CO_2_ evolution, which occurs concomitantly with the release of the polyol (Scheme 1). Surprisingly, acidolysis reactions with both acids proceeded to completion at 140 °C, far below the melting point of SA and PA (*T*_m_ = 186 and 207 °C, respectively). Figure 1 compares the time required for acidolysis with SA and PA at 140 °C. SA required ∼4.5 h (270 min), while PA required ∼2.5 h (150 min). To the best of our knowledge, PU acidolysis with DCAs at 140 °C is the lowest reaction temperature that has been reported, and further PU acidolysis below the *T*_m_ of the DCA has not been reported.

**Figure 1:**
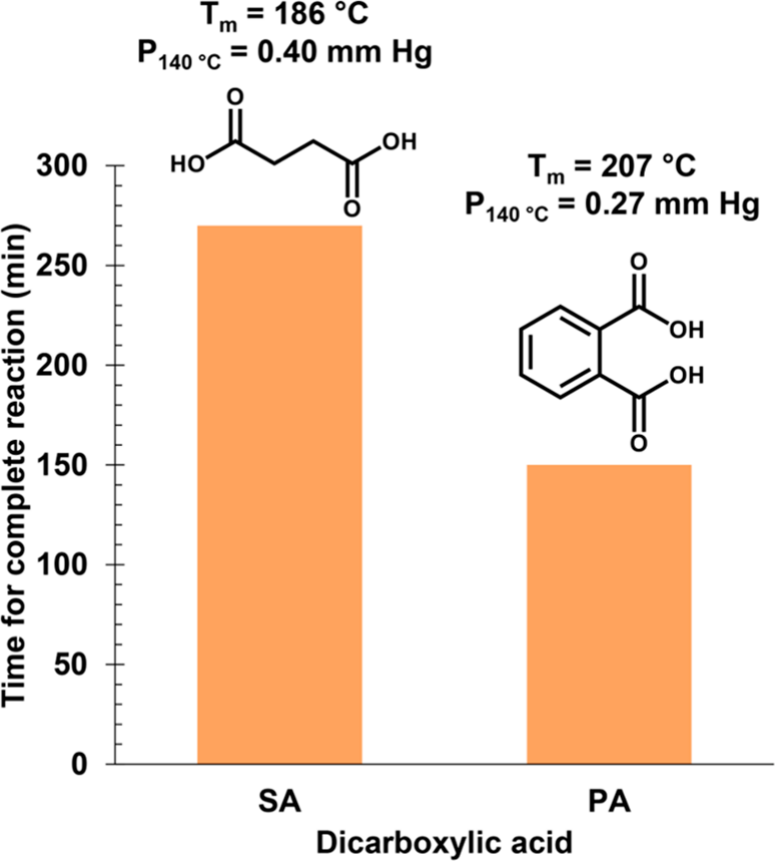
Reaction time required for acidolysis with SA and PA at 140 °C based on cessation of gas (CO_2_) evolution. Melting temperatures (*T*_m_) and vapor pressures at 140 °C (*P*_140°C_) of the DCAs are also shown.

The slow rate of solid–solid diffusion and the crystalline structure of DCAs (SA and PA) make solid-state PU acidolysis (reactions at the interface between PU and DCA solids) unlikely. However, the close contact between DCAs and ground PUF used in the experiments in Figure 1 leaves open the possibility of the inherent moisture in PUF (∼3 wt %) facilitating liquid-phase DCA transport. To test unambiguously whether acidolysis could proceed by vapor-phase transport of the DCAs, we employed a reaction setup in which the solid reactants were physically separated. In a typical reaction, several chunks of model PUF were placed in a beaker and a vial containing DCA was placed in the center, allowing physical segregation of the solid reactants (Figure 2(a)). The beaker was sealed and placed in an oven at 160 °C, along with a chunk of PUF in a separate beaker (see the SI for details). After 10 h of reaction, the model PUF was decomposed completely, while DCA remained as a solid in its vial (Figure 2(b)). In contrast, the reference PUF sample remained intact with a slight discoloration to yellow. Figure S-2 compares the TGA (thermogravimetric analysis) of a fresh model PUF and the reference PUF sample, confirming that the reference PUF sample did not undergo thermal degradation or decomposition at 160 °C. Thus, the decomposed PUF in our physically separated reaction setup resulted from acidolysis by vapor-phase transport of DCA (or DCA derived species).

**Figure 2:**
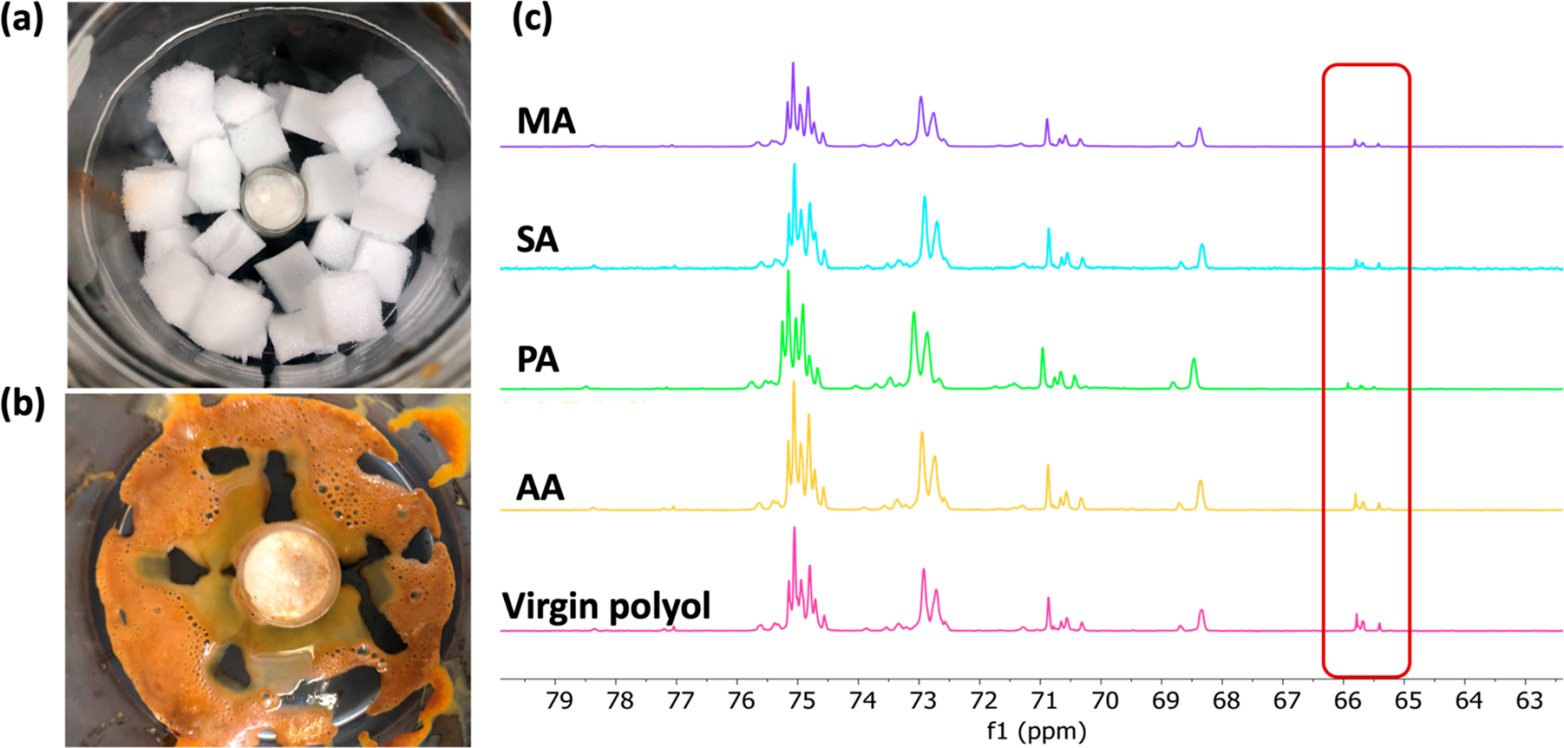
Depiction of the vapor-phase acidolysis setup with physically separated PUF (5 g) and DCA (2 g) (a) before and (b) after reaction at 160 °C. (c) ^13^C NMR (nuclear magnetic resonance) of polyol recovered from vapor-phase acidolysis reactions with different DCAs. The signals from the −OH end groups of the polyol were retained, indicating that esterification of polyol was partially prevented during the vapor-phase acidolysis reaction.

Figure 2(c) shows the ^13^C NMR spectra of polyol obtained from vapor-phase (from the physically segregated reaction) acidolysis of model PUF with several DCAs. The signal of the carbon backbone of each recovered polyol was identical to the virgin polyol (VORANOL 8136). More importantly, compared to the polyol obtained from liquid-phase acidolysis with direct contact between model PUF and solid/liquid DCA (Figure S-1), the signal of the −OH end group was observed (δ ^13^C 65–66.7) for the polyol product from the reactions with acid vapor, suggesting the esterification side reaction between DCA and polyol was partially prevented (Scheme S-2). Additionally, PUF acidolysis driven by acid vapor does not require pregrinding of the foam or mechanical mixing (Figure S-3). Finally, vapor-phase acidolysis of EOL PUF was also successful with SA at 160 °C (Figure S-4), indicating its viability for use on postconsumer PU products.

During acidolysis with MA and PA, the formation of excess water (beyond what is expected from the decomposition of urea bonds) was observed. DCAs dehydrate upon heating to form acid anhydrides, which have higher vapor pressures and lower *T*_m_ than their respective acids (Table S-1).^8^ Heating of MA and PA in the absence of PUF also produced water, confirming that anhydrides were formed at temperatures at which acidolysis reactions were executed. Reactions between anhydrides and carbamates/polyurethanes have been reported, but it is unclear how an anhydride could break the C–O or C–N bond of urethane or urea without first hydrolyzing to an acid.^9,10^ However, the high volatility of anhydrides compared to acids may allow them to transport in the vapor phase more effectively than the acids. TGA measurements demonstrate that the model PUF retains ∼3% moisture content after shredding and drying. This moisture content is sufficient to hydrolyze the required anhydride to fully decompose the PUF (based on the moles of urea and urethane bonds), suggesting that DCA anhydrides may act as a mobile but nonreactive species.

To assess the role of corresponding anhydrides of DCAs in this process, succinic anhydride (SAnh) and phthalic anhydride (PAnh) were tested directly for their role in vapor-phase PU acidolysis at 180 °C. Model PUF was sealed in a graduated bottle with a calcination boat containing acid or anhydride and placed in an oven for 4 h; the experiment was then repeated with 325 μL of water added (Figures S-5–S-8). Figure 3 shows the first 90 min of reaction of PU with PA versus PAnh. Both PA and PAnh were able to decompose the PUF foam; however, the rate of acidolysis with PA was slower than that with PAnh, suggesting that PAnh vapor more effectively transports to the PUF foam than PA. Furthermore, the addition of water accelerated acidolysis with both PA and PAnh. This indicates that hydrolysis of PAnh to PA is required to initiate the acidolysis reaction. The increased rate of acidolysis observed for PA with the addition of water suggests that transport of PA to the foam surface is facilitated by the acid-anhydride equilibrium, with PA dehydrating to PAnh, transporting in the vapor phase, then subsequently hydrolyzing back to PA to induce acidolysis. The amount of added water (325 μL) is 10-fold larger than the amount of water that is inherent to the foam surface (∼3 wt % or 30 μL on 1 g of PUF). It is hypothesized that the additional water shifts the equilibrium of the acid-anhydride reaction, thereby increasing the concentration of PA near the surface of the foam. We note that observed recondensation in the reaction vessel demonstrates that vapor did not appreciably leave the system. Therefore, we propose that PAnh serves as the predominant mobile species for vapor-phase PUF acidolysis, while PA serves as the reactive species at the PU surface following rehydration of PAnh. Similar results were obtained for vapor-phase acidolysis with SA and SAnh, although SA appears to be more mobile than PA under otherwise identical conditions, which is expected given their relative volatilities (Figures S-7 and S-9).

**Figure 3:**
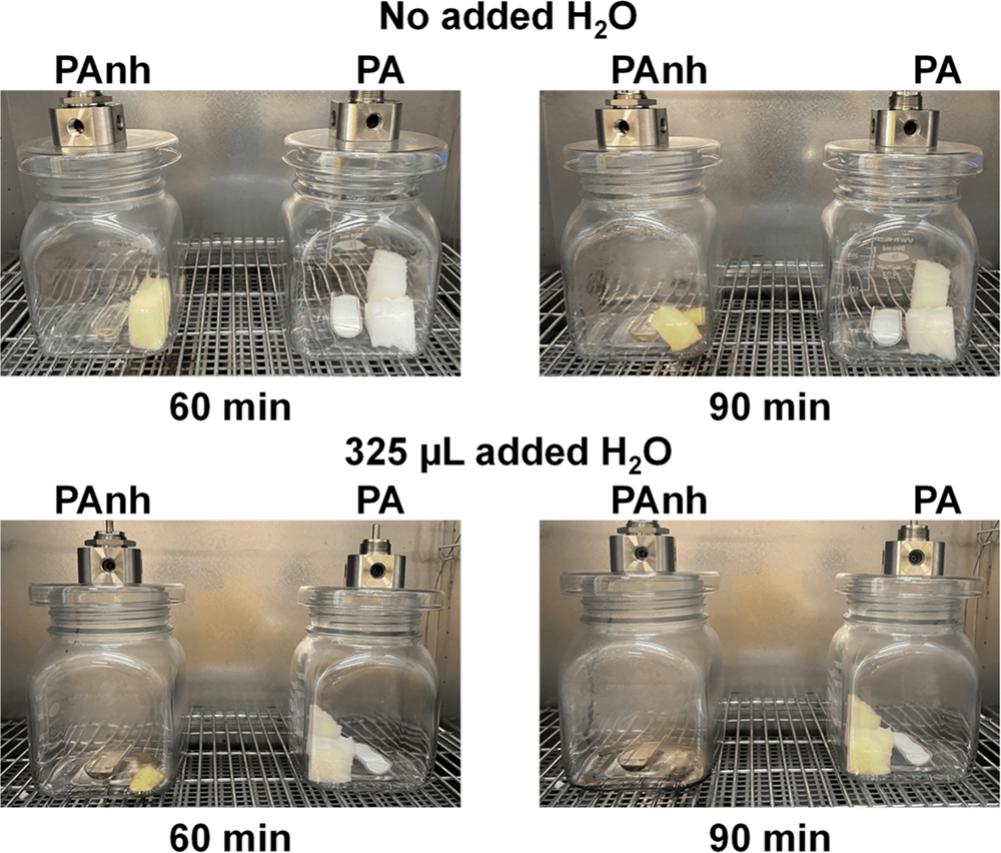
Photos of a vapor-phase acidolysis reactor setup comparing acidolysis of PUF (∼1 g) with 1.5 g of phthalic acid (PA) or phthalic anhydride (PAnh), with and without the presence of added water at various reaction times.

To determine whether PAnh was capable of decomposing urethane bonds with no added H_2_O (i.e., serve also as the reactive species), PA and PAnh acidolysis reactions were studied with a model urethane, *tert*-butyl *N*-(pyridine-4-yl)-carbamate (4-tBuCAP). These reactions were conducted by physically mixing the reactants in a round-bottom flask and heating to 120 °C in an oil bath; the CO_2_ evolution was monitored using a gas evolution buret. The model urethane (4-tBuCAP) contains no moisture, and the flask was loaded in a glovebox to remove the potential of water facilitating PA formation (this is distinct from model PUF where water cannot be entirely removed without causing foam degradation). Figure 4 shows the ^1^H NMR spectra of the reaction mixtures after 1 h. The disappearance of signals at δ ^1^H = 10.07, 8.41, and 7.52 indicates that 4-tBuCAP was fully decomposed by PA. Signals at δ ^1^H = 8.12, 8.02, and 6.81 also indicate the formation of an amine product. In contrast, 4-tBuCAP signals remained in the ^1^H spectrum after the reaction with PAnh, and no additional product peaks were detected. Furthermore, no gas product was observed from 4-tBuCAP + PAnh reaction, indicating that PAnh did not decompose the urethane bond of 4-tBuCAP in the absence of water. Acidolysis of 4-tBuCAP with PA evolved gas, consistent with the production of CO_2_ and t-BuOH (Figure S-9).

**Figure 4:**
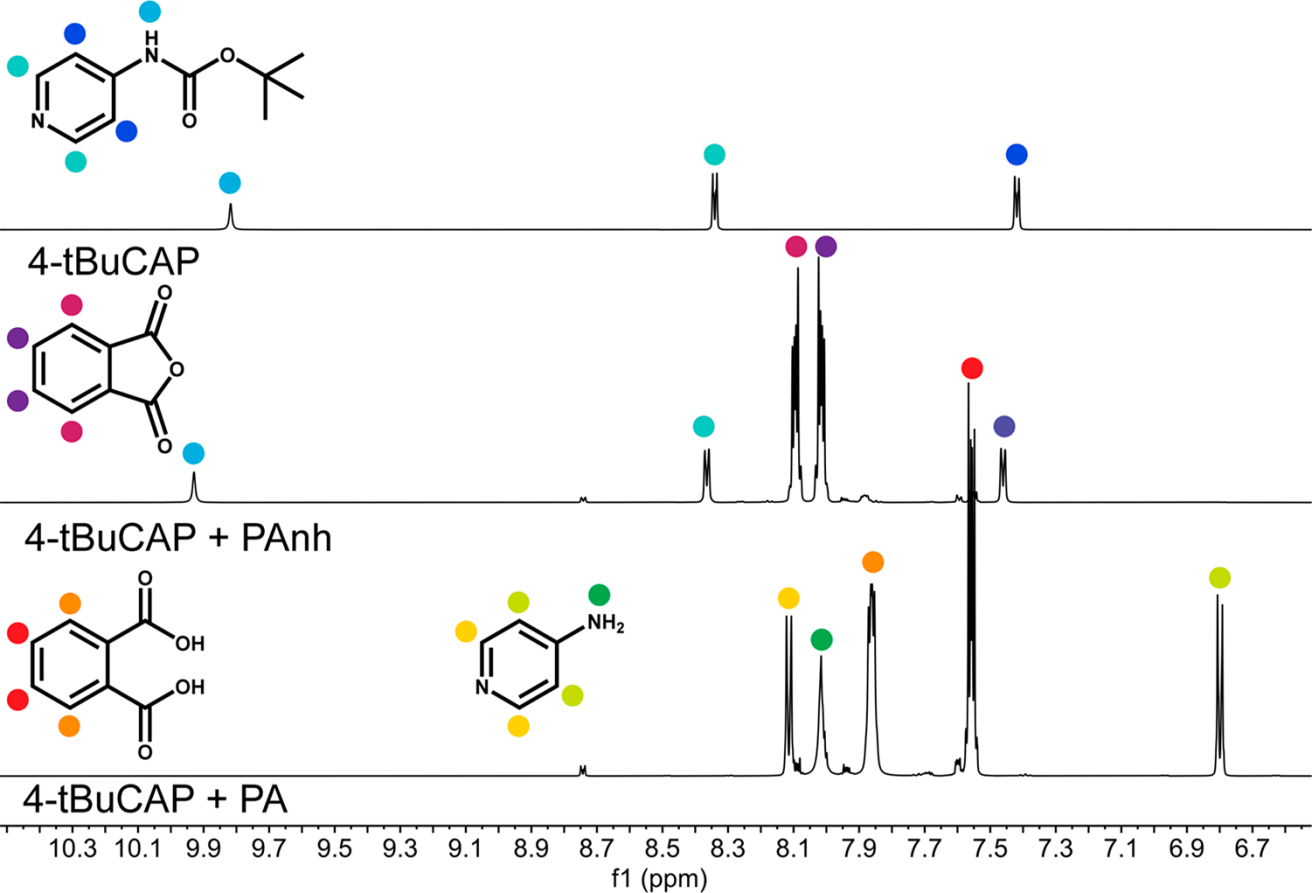
^1^H NMR spectra of the fresh model urethane 4-tBuCAP (top) and following reactions with PAnh (middle) and PA (bottom) at 120 °C for 1 h.

These results confirm that anhydrides are unreactive for PU acidolysis and must hydrolyze to the corresponding acids to decompose PUF. Thus, anhydrides, which have higher vapor pressures than their respective DCAs, can serve as the predominant mobile species to transport acid (by hydrolyzing the anhydride back to the acid at or on the PUF surface) to the foam surface under the reaction conditions. The small moisture content within PUF is therefore hypothesized to be crucial for vapor-phase PU acidolysis with acid anhydrides.

In this work, we have demonstrated closed-loop PUF chemical recycling via vapor-phase acidolysis at moderate temperatures between 140 and 180 °C, far below the melting temperature of organic diacids and temperatures typically reported in the literature. We demonstrated that anhydrides serve as mobile species but require moisture to hydrolyze back to the DCA to be reactive for PU acidolysis. The polyol obtained from the vapor-phase reaction contains free −OH end groups, allowing direct reuse without further treatment compared to the polyol ester obtained from liquid-phase acidolysis. Both model PUF and EOL (end-of-life) PUF waste were evaluated in this work, which confirms the ability of vapor-phase acidolysis to chemically recycle EOL PUF from commercial markets. The described vapor-phase PUF acidolysis adheres to the principles of green chemistry—reduction of undesired byproducts, minimization of post reaction processing (polyol product instead of polyol ester), improved energy efficiency, no solvent, and minimization of reagent use compared to condensed-phase reaction, which requires excess organic acid. Future studies are aimed at obtaining kinetics and understanding the reaction mechanism.

## Supporting Information Available

Materials, experimental procedures, analytical methods, chemical structures of toluene diisocyanate (TDI) and polyether polyol, reaction scheme of esterification between DCAs and repolyol, ^13^C HSQC (heteronuclear single quantum coherence) NMR spectra, results of TGA measurement, illustration of vapor-phase PUF acidolysis reaction setups, vapor pressure of DCAs and anhydrides between 150–200 °C, and results of gas evolution detections (PDF)

## References

[ref1] Gadhave R. V.; Srivastava S.; Mahanwar P. A.; Gadekar P. T. Recycling and Disposal Methods for Polyurethane Waste: A Review. Open Journal of Polymer Chemistry 2019, 09 (02), 39–51. 10.4236/ojpchem.2019.92004.

[ref2] Liu B.; Westman Z.; Richardson K.; Lim D.; Stottlemyer A. L.; Farmer T.; Gillis P.; Vlcek V.; Christopher P.; Abu-Omar M. M. Opportunities in Closed-Loop Molecular Recycling of End-of-Life Polyurethane. ACS Sustainable Chem. Eng. 2023, 11 (16), 6114–6128. 10.1021/acssuschemeng.2c07422. PMC10952008 38516400

[ref3] Gama N.; Godinho B.; Marques G.; Silva R.; Barros-Timmons A.; Ferreira A. Recycling of polyurethane scraps via acidolysis. Chem. Eng. J. 2020, 395, 125102 10.1016/j.cej.2020.125102.

[ref4] Gama N.; Godinho B.; Marques G.; Silva R.; Barros-Timmons A.; Ferreira A. Recycling of polyurethane by acidolysis: The effect of reaction conditions on the properties of the recovered polyol. Polymer 2021, 219, 123561 10.1016/j.polymer.2021.123561.

[ref5] Godinho B.; Gama N.; Barros-Timmons A.; Ferreira A. Recycling of polyurethane wastes using different carboxylic acids via acidolysis to produce wood adhesives. J. Polym. Sci. 2021, 59 (8), 697–705. 10.1002/pol.20210066.

[ref6] Grdadolnik M.; Drincic A.; Oreski A.; Onder O. C.; Utrosa P.; Pahovnik D.; Zagar E. Insight into Chemical Recycling of Flexible Polyurethane Foams by Acidolysis.. ACS Sustain Chem. Eng. 2022, 10 (3), 1323–1332. 10.1021/acssuschemeng.1c07911. 35096493 PMC8790754

[ref7] Liu B.; Westman Z.; Richardson K.; Lim D.; Stottlemyer A. L.; Farmer T.; Gillis P.; Hooshyar N.; Vlcek V.; Christopher P.; Abu-Omar M. M. Polyurethane Foam Chemical Recycling: Fast Acidolysis with Maleic Acid and Full Recovery of Polyol.. ACS Sustainable Chem. Eng. 2024, 12 (11), 4435–4443. 10.1021/acssuschemeng.3c07040. 38516400 PMC10952008

[ref8] Yaws C. L.; Narasimhan P.; Gabbula C. Yaws’ Handbook of Antoine Coefficients for Vapor Pressure, 2nd Electronic ed.; Knovel (Elsevier Science): 2015. 10.1016/B978-0-12-802999-2.00002-7.

[ref9] Soltysinski M.; Piszczek K.; Romecki D.; Narozniak S.; Tomaszewska J.; Skórczewska K. Conversion of Polyurethane Technological Foam Waste and Post-Consumer Polyurethane Mattresses into Polyols - Industrial Applications. Polimery/Polymers 2018, 63 (3), 234–238. 10.14314/polimery.2018.3.8.

[ref10] Michman M.; Patai S.; Wiesel Y. Organic reactions in melts and solids. Part 10. Reactions of carboxylic acids and anhydrides with carbamates. J. Chem. Soc., Perkin Trans. 1977, 1 (15), 1705–1710. 10.1039/p19770001705.

