# Supplementary material for: Vapor-Phase Dicarboxylic Acids and Anhydrides Drive Depolymerization of Polyurethanes
Source: ACS Macro Lett. 2024 Mar 28;13(4):435–9. doi: 10.1021/acsmacrolett.4c00008 (PMC11025130; doi:10.1021/acsmacrolett.4c00008)
Supplement: Supplementary file 1 — mz4c00008_si_001.pdf [file mz4c00008_si_001.pdf]

Supporting Information

Vapor Phase Dicarboxylic Acids and Anhydrides Drive Depolymerization of Polyurethanes

Baoyuan Liu,[a†] Zach Westman,[b†] Kelsey Richardson,[b] Dingyuan Lim,[a] Alan Stottlemyer,[c] Paul Gillis,[c] Christopher S. Letko,[c] Nasim Hooshyar,[d] Vojtech Vlcek,[a] Phillip Christopher,[b,*] and Mahdi M. Abu-Omar[a,b,*]

[a] *Department of Chemistry and Biochemistry, University of California, Santa Barbara, CA, United States 93117.*

[b] *Department of Chemical Engineering, University of California, Santa Barbara, CA, United States 93117.*

[c] *The Dow Chemical Company, Midland, MI, United States 48642.*

[d] *The Dow Chemical Company, Herbert H Dowweg 5, Hoek 4542 NH, The Netherlands*

* *Correspondence to: pchristopher@ucsb.edu , mabuomar@ucsb.edu*

† These authors contributed equally to this work and share first authorship.

Table of Content:

Page 2-4: Material and Experimental Procedure

Page 5-13: Figures and Scheme

Page 5: Scheme S-1 Chemical structures of TDI and polyether polyol

Page 6: Figure S-1 ^{13}C HSQC NMR of virgin polyol, repolyol, and amide products

Scheme S-2 Illustration of esterification reaction between DCA and repolyol

Page 7: Figure S-2 TGA measurements of intact model PUF and reference model PUF

Page 8: Figure S-3 Scale-up reaction of contactless acidolysis

Figure S-4: Illustration of EOL PUF acidolysis performed in a contactless setup

Page 9: Table S-1 Vapor pressure of selected DCAs and their anhydrides

Figure S-5 Vapor-phase PUF acidolysis with PAnh and PA without water

Page 10: Figure S-6 Vapor-phase PUF acidolysis with PAnh and PA with 325 μL water

Page 11: Figure S-7 Vapor-phase PUF acidolysis with SAnh and SA without water

Page 12: Figure S-8 Vapor-phase PUF acidolysis with SAnh and SA with 325 μL water

Page 13: Figure S-9 Gas evolution for PUF acidolysis with PA and PAnh with 4-tBuCAP

Material and Experimental Procedure

The model PUF sample was provided by The Dow Chemical Company (Dow) which was synthesized from VORANOL™ 8316 polyether polyol (the virgin polyol, 71 wt% of the model PUF) and VORANATE™ T-80 toluene diisocyanate (TDI). The model PUF used for this study was an open cell flexible foam. The commercial end-of-life PUF (EOL PUF) waste was provided by Dr. Nasim Hooshyar from Dow in Europe. The EOL PUF was a random mixture of mattress waste of which the type and content of polyol used in the EOL PUF were unknown.

Maleic acid, succinic acid, phthalic acid, adipic acid and their corresponding acid anhydrides were purchased from Sigma-Aldrich and used as is. The acid anhydrides were stored under argon atmosphere in glovebox. Calcium oxide (CaO) purchased from Sigma-Aldrich was used to prepare the $Ca(OH)_2$ solution to quantify the CO_2 products. Ethyl acetate (EtOAc, ACS regent) was purchased from Sigma-Aldrich. NMR solvent Hexadeuterated dimethyl sulfoxide (DMSO-d_6) was purchased from Cambridge Isotope Laboratories Inc. High purity nitrogen gas (N_2, 5.0 grade) was purchased from Praxair Technology Inc.

Grinding Pre-treatment of Model and EOL PUF. Prior to the acidolysis reaction, both model PUF and EOL PUF chunks were grinded into smaller particles. The foam chunks were first flask frozen in liquid nitrogen to increase their brittleness and transferred subsequently into a mechanical grinder equipped with cross blades. After grinding, the particle size of model PUF was between 500 – 2000 μm, while the EOL PUF particles were around 155 – 750 μm.

PUF Acidolysis Reaction Setup with Direct Contact of Acid. The contact acidolysis reaction was carried out in a glass reaction system. In this setup, a 250 mL round bottom flask was connected to a cold finger that was connected through Tygon tubing to a gas evolution burette.

The DCA was mixed with the shredded PUF in the round bottom flask. After that, the whole system was purged with N_2 to eliminate air and moisture and 1 atm N_2 was left in the reaction system. In contrast, the PUF acidolysis with DCA anhydride was carried out in a 100 mL airtight pear-shaped Schlenk flask tube. The shredded PUF and DCA anhydride were loaded to the Schlenk flask in glovebox protected by argon and sealed. The reaction mixture was heated in oil bath to the desired temperature. The reaction mixture was stirred through the whole reaction duration by magnetic stirring at 250 rpm. Completion of reaction was determined by full homogenization of the reaction mixture and cessation of gas evolution. After reaction, the product mixture was cooled to room temperature. The liquid product mixture was washed by EtOAc and vacuum filtered through a Buchner funnel. The products were dissolved in EtOAc filtrate. After removal of EtOAc solvent, the neat product mixture was dried for further analysis.

Vapor-phase PUF Acidolysis Reaction Setup. The vapor-phase PUF acidolysis setup was set up in a glass graduated bottle or beaker. Several chunks of PUF (ca. 1 g for small-scale, ca. 5-25 g for scaled-up reaction, chunk size 1 - 10 cm^3) were placed at the bottom of the bottle. A vial or calcination boat containing DCA or anhydride was placed in the center of the bottle, ensuring no physical contact between solid acid/anhydride and PUF. In experiments comparing acid and anhydride, 325 μL of HPLC H_2O (approximately the amount produced by complete dehydration of 3 g DCA to anhydride) was added at the bottom of the bottle, taking care to ensure liquid water was not in contact with the acid/anhydride or PUF. The bottle was sealed with a watch glass and placed in an oven precalibrated to the desired temperature (160 – 180 °C). Reactions were run for 4 – 10 hours; for acid/anhydride comparisons, the temperature of the oven and the progression of the reaction was monitored every 30 min.

Thermogravimetric Analysis (TGA) of PUF Samples. Discovery 5500 Thermo-Gravimetric Analyzer was used for TGA analysis of PUF samples. For each measurement, 5 – 10 mg of foam samples was loaded to an alumina ceramic crucible. The crucible was then placed on a high temperature platinum sample pan which was calibrated prior to each measurement. The TGA analysis was performed under 25 mL/min N_2 flow. For each measurement, the TGA chamber was first heated to 50 ℃ and held for 5 min to determine the moisture content within the PUF sample. After that, the PUF sample was heated to 550 ℃ at a ramping rate of 20 ℃/min. The weight change between 220 – 320 ℃ was assigned to the urea bond decomposition, while the weight change between 320 – 450 ℃ was assigned to the thermal decomposition of urethane bonds.

^{13}C HSQC Nuclear Magnetic Resonance Spectroscopy (NMR) of Products from PUF Acidolysis. The ^{13}C NMR analysis was carried out with a Bruker Avance NEO 500 MHz spectrometer which equipped with a 5 mm X-nuclei optimized double resonance cryoprobe. For each measurement, 100 – 150 mg sample was dissolved in 600 μL DMSO-d_6 and packed in 5 mm NMR tube. The chemical shifts of the polyol were assigned according to the internal information share by Dow. The key assignments, such as the ^{13}C chemical shifts (δ^{13}C) of carbon with hydroxyl (OH) ending group on polyol, were between 65 – 66.7 ppm. The δ^{13}C between 67.5 – 79.5 ppm are the major carbon backbones of the polyether polyol. The δ^{13}C between 110 – 140 ppm and 160 – 170 ppm are assigned to the amide carbons. The δ^{13}C of PA and PA anhydride assignments are based on the pure standards of PA and PA anhydride.

Figures and Scheme

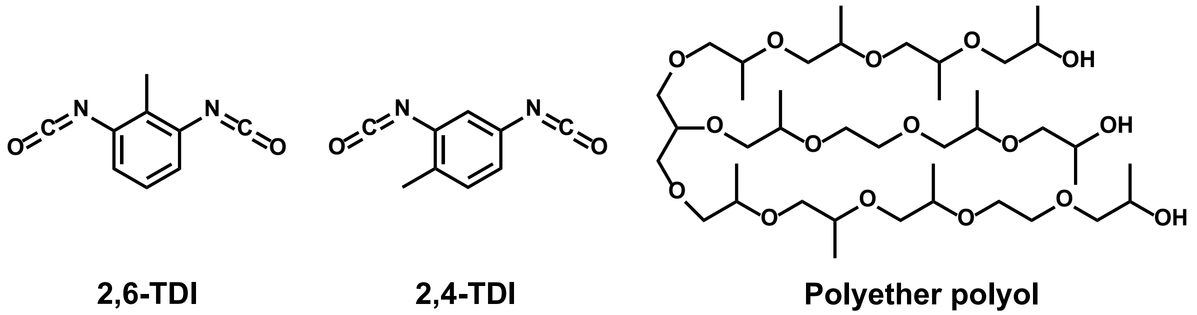

Scheme S-1. Chemical structure of 2,6-toluene diisocyanate (2,6-TDI), 2,4-toluene diisocyanate (2,4-TDI), and a representative of virgin polyether polyol used for synthesizing model PUF.

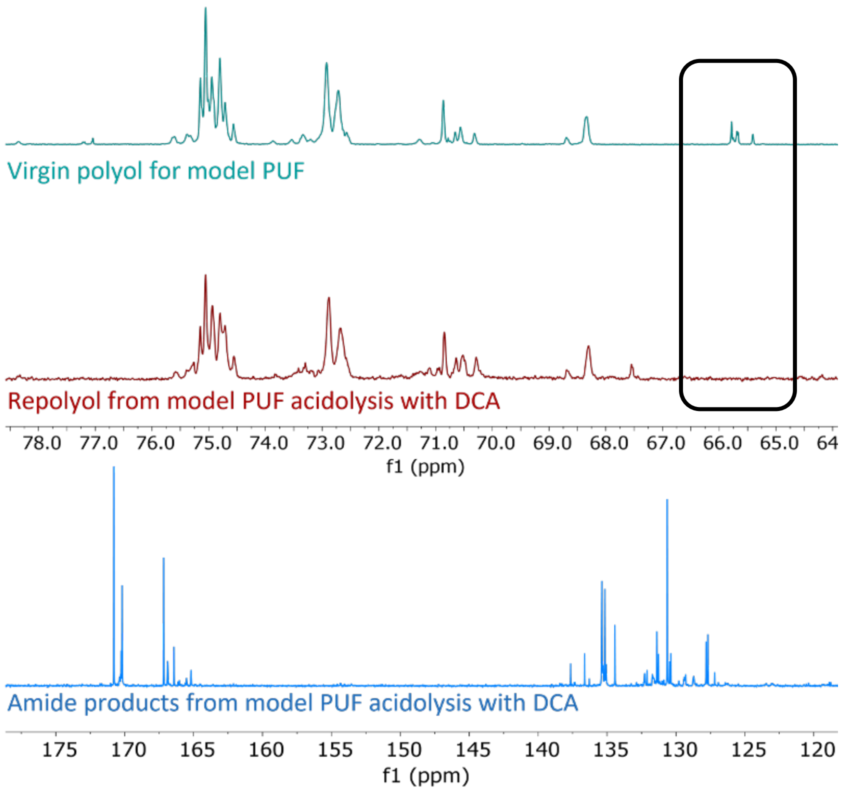

Figure S-1. ^{13}C HSQC NMR spectra of virgin polyol used to synthesize model PUF, repolyol obtained from model PUF acidolysis with MA at 175 °C, and amide products. Notably, the hydroxyl end groups (boxed, between δ ^{13}C 65 – 66.7 ppm) of the resulting repolyol were not shown after acidolysis reaction.

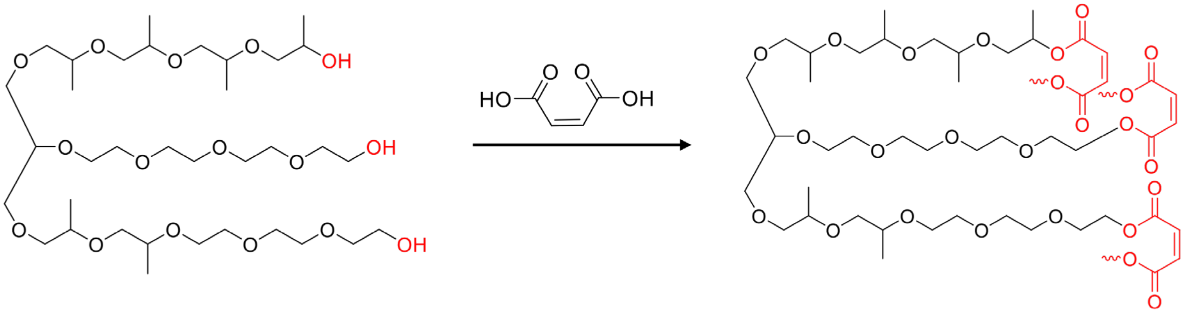

Scheme S-2. Illustration of esterification reaction between DCA (example of MA) and repolyol. The hydroxyl (OH) group on repolyol was reacted with the carboxylic acid group (COOH) from MA. The signal of $\delta^{13}C$ between 65 – 66.7 ppm on ^{13}C NMR spectrum for C-OH detection was not observed for repolyol that obtained from acidolysis reaction between model PUF and DCA in the reaction setup with direct contact.

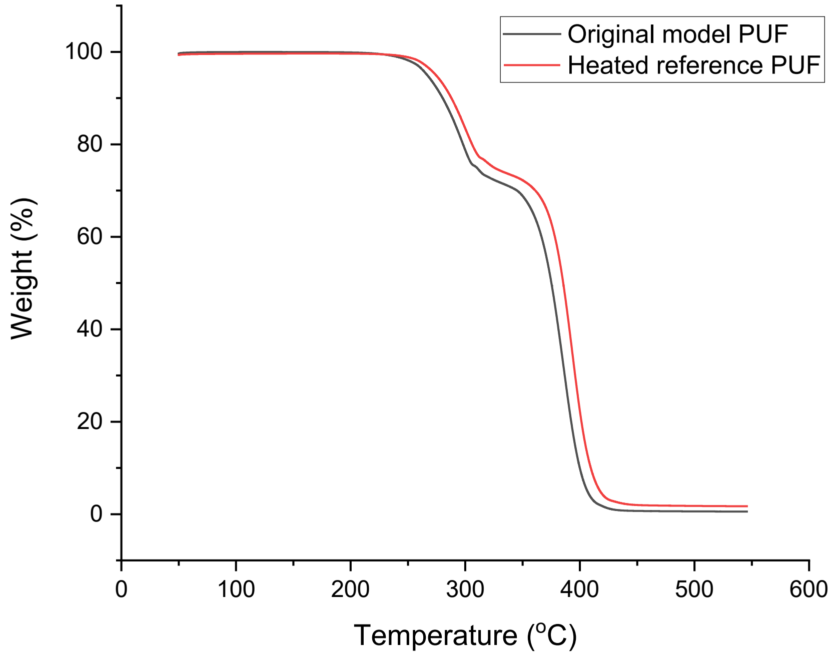

Figure S-2. TGA measurement of the intact model PUF (black color) and the reference model PUF (red color) heated at 160 °C under air over 8 h.

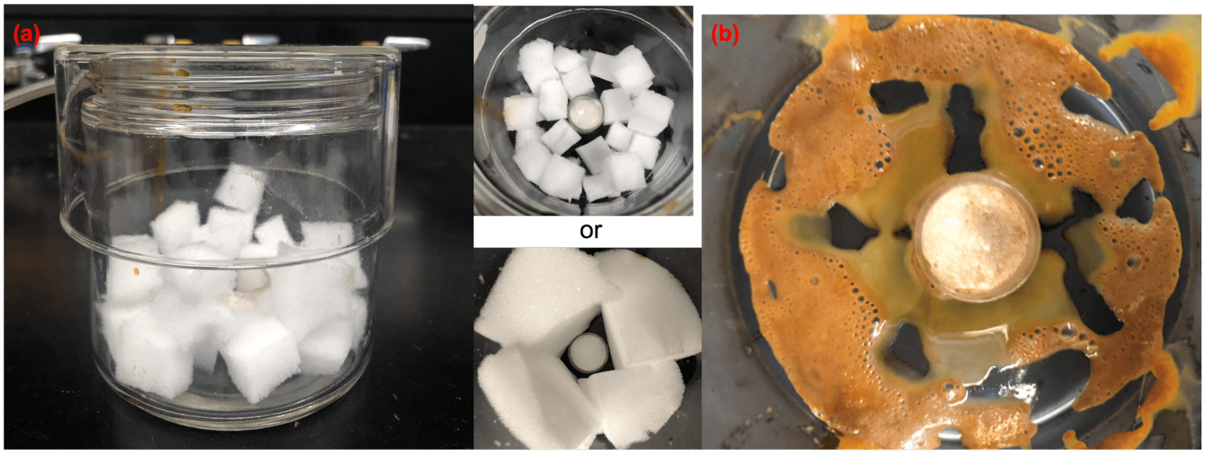

Figure S-3. The scale-up reaction of contactless acidolysis. (a) Small or large PUF chunks (up to 25 g) could be fitted into a reaction chamber. The solid DCA was isolated in a small beater in the middle of the reaction chamber. The reaction chamber was covered and heated in a conventional oven to desired temperature. (b) After reaction, the PUF chunks were decomposed into liquid product, while the solid DCA stayed contactless from the foam products in the beaker.

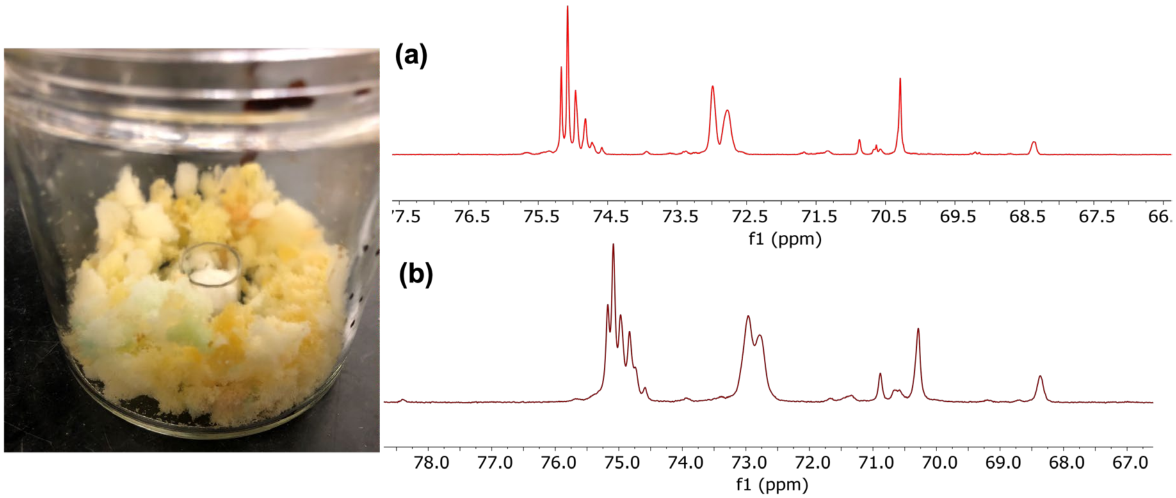

Figure S-4. Illustration of EOL PUF acidolysis performed in a contactless setup. ^{13}C NMR spectra compare the repolyol products of EOL PUF obtained from different reaction condition: (a) EOL PUF was physically mixed with SA in a round bottom flask. The acidolysis reaction was performed at 190 °C for 30 min. (b) Acidolysis reaction of EOL PUF with SA was carried out in the contactless reaction setup where the SA solid was isolated from the EOL PUF in a beaker. The reaction was performed at 160 °C overnight. As a result, the repolyol product obtained from both reaction setups and conditions gave the same chemical shifts on ^{13}C NMR. This suggests the contactless acidolysis strategy can be applied to recycle real PUF waste from the market.

	Vapor pressure (mm Hg)			
**Temperature (°C)**	**Succinic acid**	**Phthalic acid**	**Succinic anhydride**	**Phthalic anhydride**
150	0.79	0.54	23.68	8.00
160	1.46	1.03	34.81	12.62
170	2.61	1.88	50.21	19.41
180	4.51	3.29	71.18	29.20
190	7.54	5.58	99.26	43.02
200	12.23	9.17	136.33	62.16

Table S-1. Vapor pressures of selected DCAs and their anhydrides from 150 – 200 °C. Vapor pressures were determined by extracting Antoine's equation parameters from experimental data.[7]

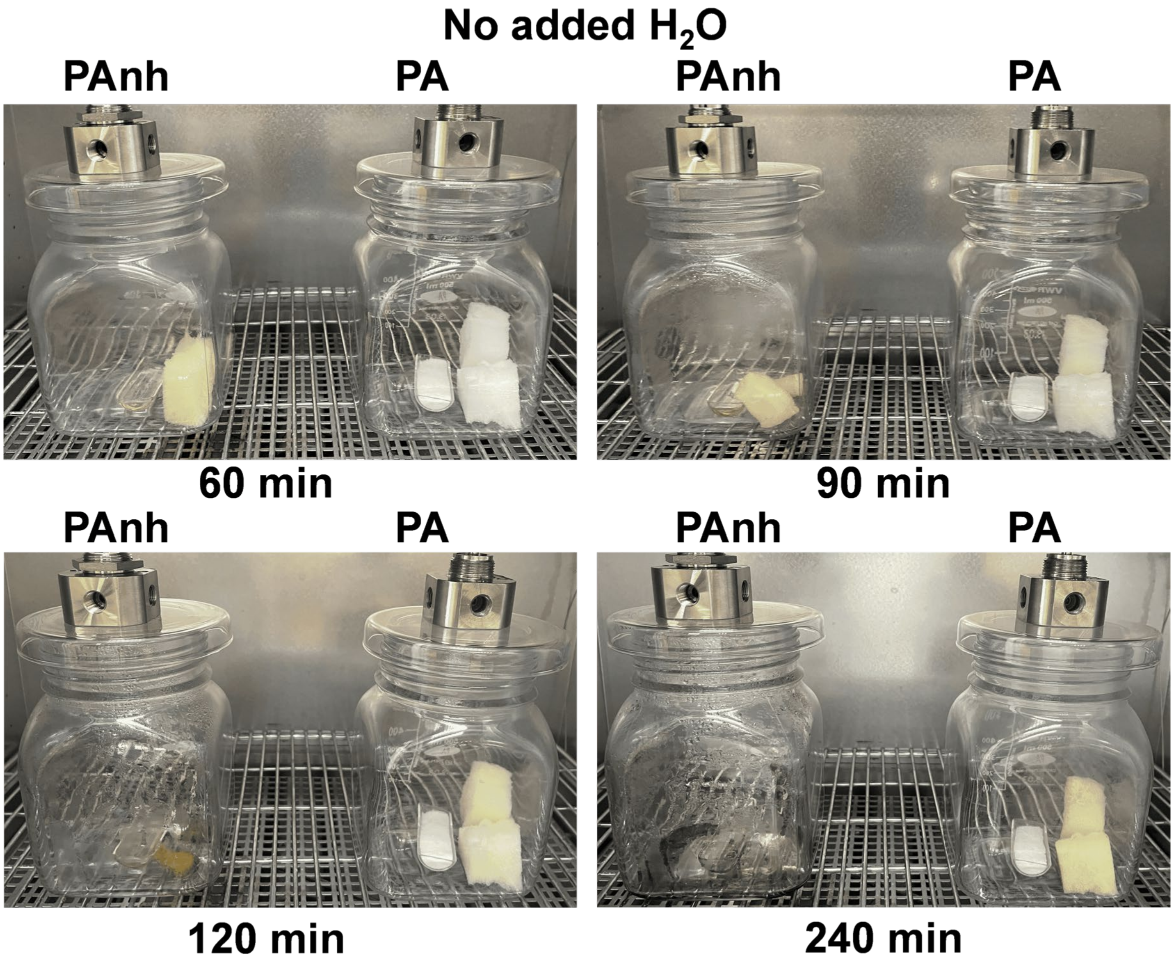

Figure S-5. Vapor-phase PUF acidolysis with PAnh and PA (2 g) and no added water. PA and PAnh were placed in calcination boats, then put in 500 mL graduated bottles with 2 chunks of PUF (~1 g) and sealed. Bottles were placed in an oven at 180 °C for four hours and progress of reaction was checked at 60, 90, 120 and 240 min.

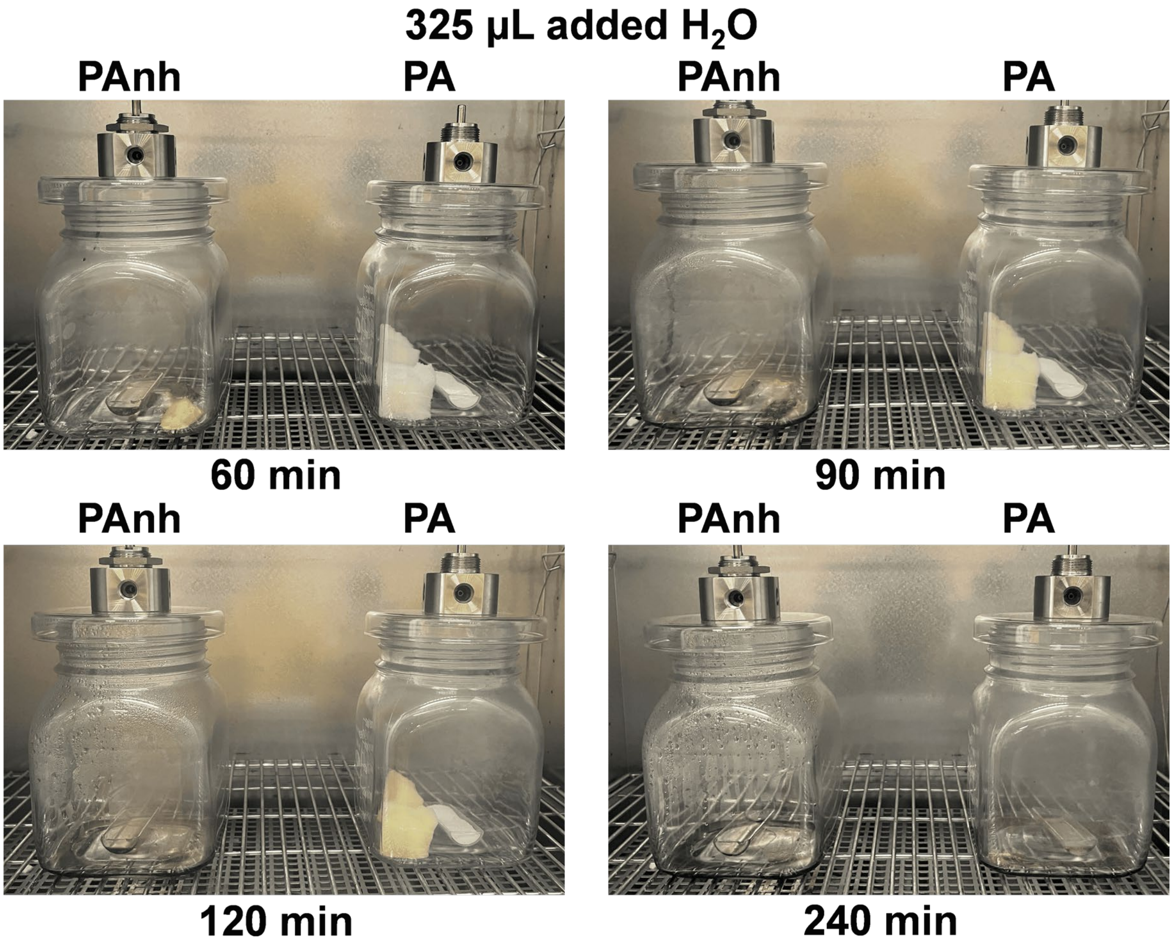

Figure S-6. Vapor-phase PUF acidolysis with PAnh and PA (2 g) and 325 µL added water. PA and PAnh were placed in calcination boats, then put in 500 mL graduated bottles with 2 chunks of PUF (~1 g) and sealed. Bottles were placed in an oven at 180 °C for four hours and progress of reaction was checked at 60, 90, 120 and 240 min.

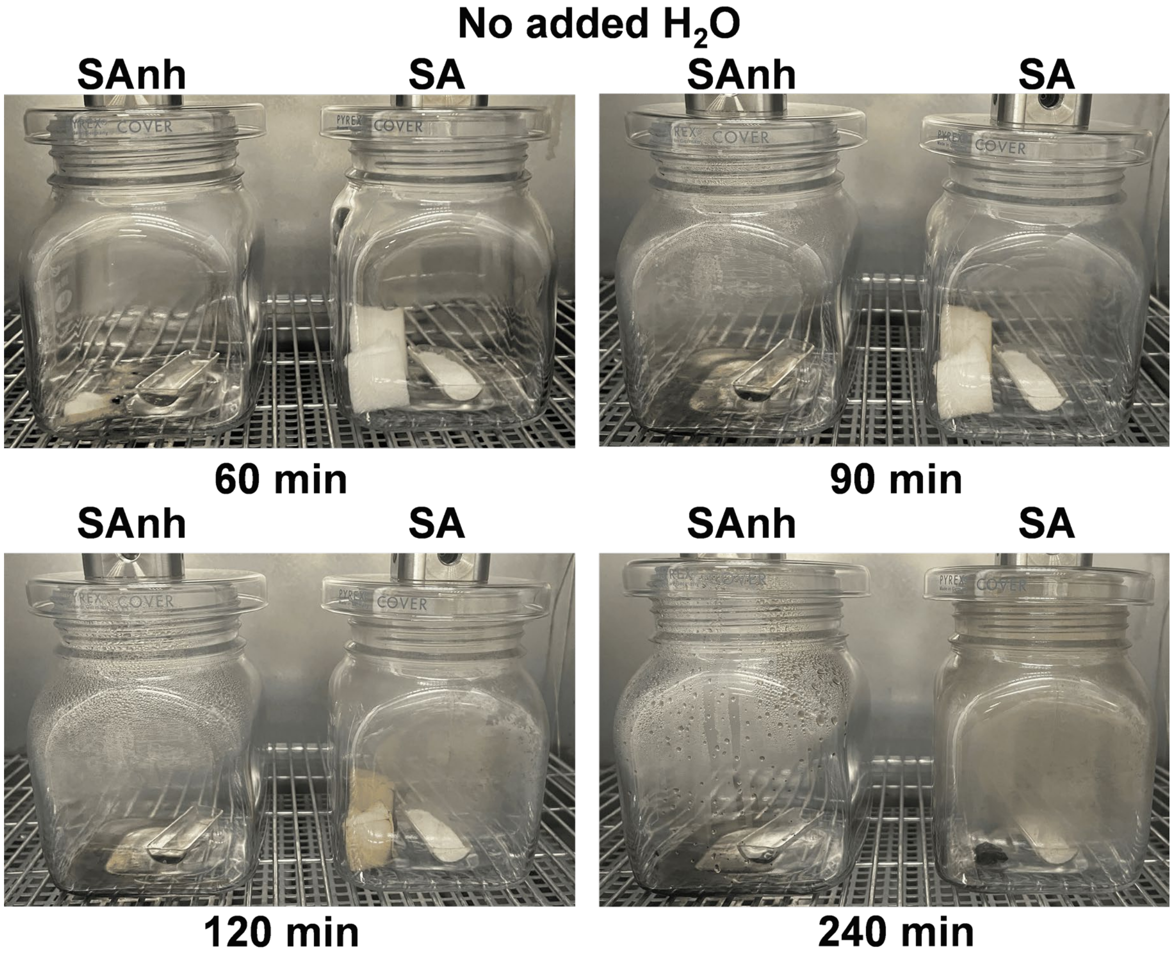

Figure S-7. Vapor-phase PUF acidolysis with SAnh and SA (2 g) and no added water. SAnh and SA were placed in calcination boats, then put in 500 mL graduated bottles with 2 chunks of PUF (~1 g) and sealed. Bottles were placed in an oven at 180 °C for four hours and progress of reaction was checked at 60, 90, 120 and 240 min.

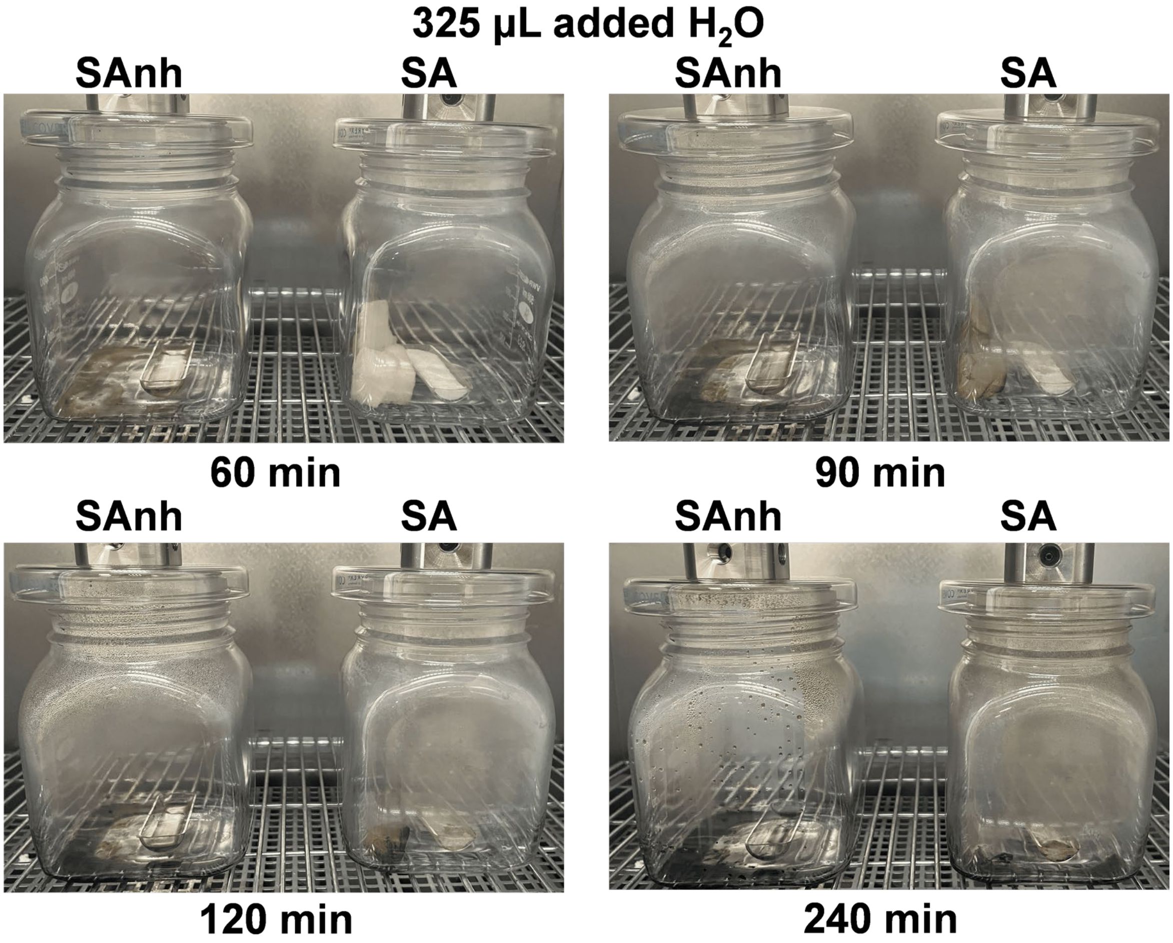

Figure S-8. Vapor-phase PUF acidolysis with SAnh and SA (2 g) and 325 μL added water. SAnh and SA were placed in calcination boats, then put in 500 mL graduated bottles with 2 chunks of PUF (~1 g) and sealed. Bottles were placed in an oven at 180 °C for four hours and progress of reaction was checked at 60, 90, 120 and 240 min.

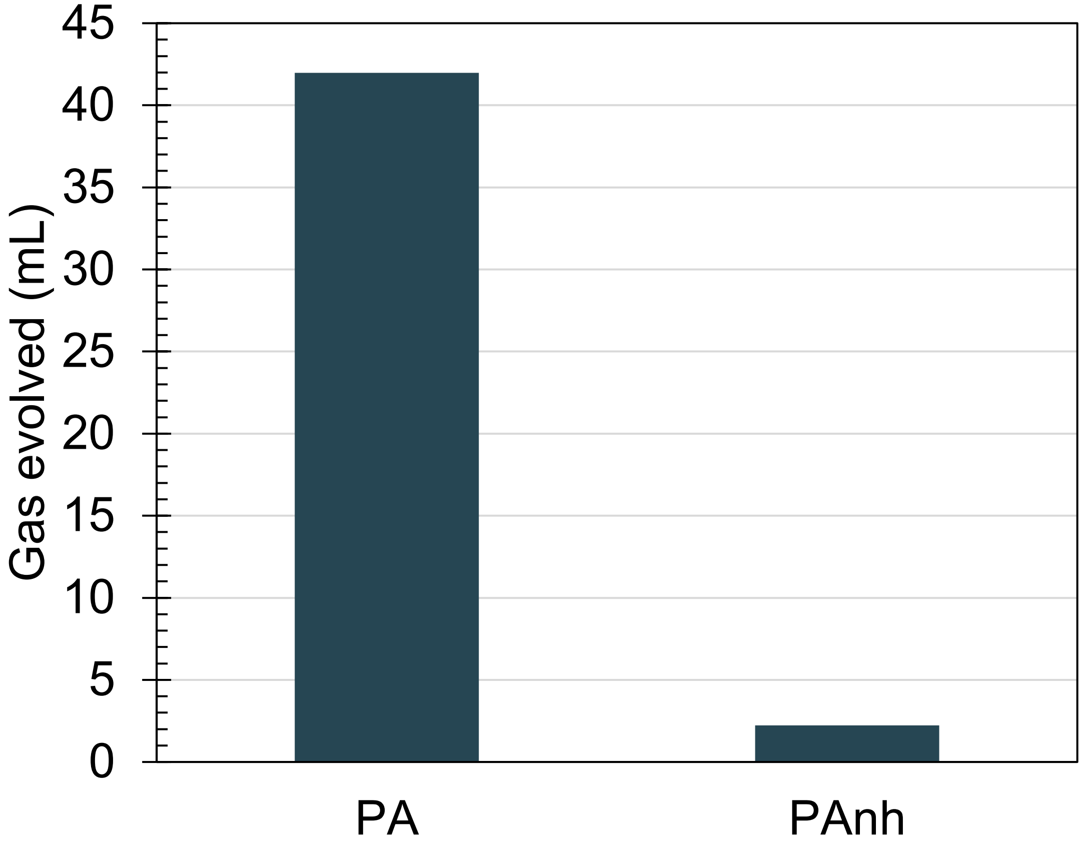

Figure S-9. Gas evolution for the reactions of 800 mg PA and PAnh with 200 mg 4-tBuCAP at 120 °C. Gas evolution with PA indicated decomposition of 4-tBuCAP, while no reaction was observed between PAnh and 4-tBuCAP.
